# Cardiovascular outcomes and mortality after initiation of canagliflozin: Analyses from the EASEL Study

**DOI:** 10.1002/edm2.96

**Published:** 2019-10-15

**Authors:** Jacob A. Udell, Zhong Yuan, Patrick Ryan, Toni Rush, Nicholas M. Sicignano, Michael Galitz, Norman Rosenthal

**Affiliations:** ^1^ Department of Medicine Cardiovascular Division Peter Munk Cardiac Centre Toronto General Hospital and Women’s College Hospital University of Toronto Toronto ON Canada; ^2^ Janssen Research & Development, LLC Titusville NJ USA; ^3^ Health ResearchTx, LLC Trevose PA USA; ^4^ Naval Medical Center Portsmouth VA USA; ^5^ Janssen Research & Development, LLC Raritan NJ USA

**Keywords:** major adverse cardiovascular event, sodium glucose co‐transporter 2 inhibitor, type 2 diabetes mellitus

## Abstract

**Introduction:**

In the EASEL study of patients with type 2 diabetes and high cardiovascular risk, initiation of sodium glucose co‐transporter 2 inhibitors (SGLT2i) was associated with lower risk of cardiovascular events and mortality and higher risk of below‐knee lower extremity (BKLE) amputation versus non‐SGLT2i therapies. This analysis further examined risk of cardiovascular events, cardiovascular and noncardiovascular death and BKLE amputation with the SGLT2i canagliflozin versus non‐SGLT2i.

**Methods:**

New user cohorts were constructed from Department of Defense Military Health System patients initiating canagliflozin or non‐SGLT2i (4/1/2013‐12/31/2016). Propensity score matching (1:1) controlled for imbalances in baseline covariates. Incidence rates, hazard ratios and 95% confidence intervals for time to first composite outcome of all‐cause mortality (ACM) and hospitalization for heart failure (HHF), composite major adverse cardiovascular events (MACE) and individual components were evaluated using conditional Cox models. The National Death Index was used to differentiate cardiovascular from noncardiovascular death. The exploratory safety end‐point was BKLE amputation.

**Results:**

After propensity matching, 15 394 patients with well‐balanced baseline covariates were followed for a median of 2.03 years (intent‐to‐treat). Canagliflozin showed significant benefit for ACM and HHF (*P* < .0001), MACE (*P* = .0001), cardiovascular death (*P* < .0001) and noncardiovascular death (*P* = .0018). No significant difference in risk of BKLE amputation was observed (*P* = .20), though few events were observed. Results were generally consistent in on‐treatment analyses.

**Conclusions:**

In this high cardiovascular risk cohort studied in routine clinical practice, canagliflozin was associated with lower risk of cardiovascular events, cardiovascular death and all‐cause mortality with no significant increase in BKLE amputation risk versus non‐SGLT2i.

## Graphical abstract

This retrospective new user cohort study examined the risk of cardiovascular events, cardiovascular and noncardiovascular death and below‐knee lower extremity amputation with the sodium glucose co‐transporter 2 inhibitor canagliflozin versus non‐sodium glucose co‐transporter 2 inhibitors in 15 394 propensity‐score matched high cardiovascular risk patients in routine clinical practice. Canagliflozin showed significant benefit for all‐cause mortality and hospitalization for heart failure (*P* < .0001), major adverse cardiovascular events (*P* = .0001), cardiovascular death (*P* < .0001) and noncardiovascular death (*P* = .0018) compared with non‐sodium glucose co‐transporter 2 inhibitors. No significant difference in risk of below‐knee lower extremity amputation was observed (*P* = .20), though few events were observed.

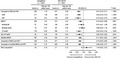

## 1 INTRODUCTION

Sodium glucose co‐transporter 2 inhibitors (SGLT2i) are a relatively new class of antihyperglycemic agents (AHAs) that increase urinary glucose excretion (ie, glycosuria) and modestly reduce circulating plasma glucose.^5^, ^29^ The EMPA‐REG OUTCOME cardiovascular (CV) outcomes trial of the SGLT2i empagliflozin in patients with type 2 diabetes mellitus (T2DM) and established CV disease has shown reductions in the primary outcome of major adverse CV events (MACE, the composite of CV death, nonfatal myocardial infarction [MI] and nonfatal stroke) and hospitalization for heart failure (HHF).^8^, ^43^ The CANagliflozin cardioVascular Assessment Study (CANVAS) Program CV outcomes trial of the SGLT2i canagliflozin in patients with T2DM and established CV disease or high CV risk also showed reductions in the primary outcome of MACE as well as HHF.^23^, ^26^ The Dapagliflozin Effect on Cardiovascular Events‐Thrombolysis in Myocardial Infarction 58 (DECLARE‐TIMI 58) CV outcomes trial of the SGLT2i dapagliflozin in patients with T2DM and established CV disease or high CV risk showed noninferiority in the co‐primary end‐point of MACE and a reduction in the second co‐primary end‐point of the composite outcome of HHF and CV death.^40^ Confirming these results, real‐world studies of SGLT2i have consistently shown CV benefits with SGLT2i in a broad range of patients with T2DM, including those with high CV risk.^14^, ^15^, ^25^, ^28^, ^38^ However, there have been concerns raised about the design of many of these pharmacoepidemiologic cohort studies, with suggestions made to improve their rigor and reduce the risk of immortal time bias, misclassification exposure bias and lead‐in time bias.^31^, ^32^

Regarding safety, below‐knee lower extremity (BKLE) amputation is a potentially serious complication of T2DM.^41^ The CANagliflozin cardioVascular Assessment Study (CANVAS) Program showed an excess risk of 3 events per 1000 patient‐years of BKLE amputation with canagliflozin in a large CV outcome trial consisting of 10 142 T2DM patients with (66%) and without (34%) established atherosclerotic CV disease followed for a mean of 3.6 years.^18^, ^24^ In contrast, an increased risk of BKLE amputation was not observed in a pooled analysis of 12 randomized controlled Phase 3 and Phase 4 clinical studies of canagliflozin in 8114 patients with T2DM with a low incidence (6.6%) of established CV disease^42^ followed for a mean of 0.9 years (data on file). Results from observational studies in T2DM patients with and without established CV disease have been mixed on the risk of BKLE amputation in patients newly initiating an SGLT2i compared with other oral diabetes therapies, glucagon‐like peptide‐1 (GLP‐1) receptor agonists and insulin.^1^, ^28^, ^38^, ^39^, ^42^

In the prior analysis of the EASEL (Evidence for Cardiovascular Outcomes With Sodium Glucose Cotransporter 2 Inhibitors in the Real World) population‐based cohort study in patients with T2DM and high CV risk, SGLT2i treatment was associated with a lower risk of all‐cause mortality (ACM), HHF, and the composite of ACM, nonfatal MI and nonfatal stroke, and a higher risk of BKLE amputation compared to treatment with a non‐SGLT2i.^38^ Patients in the prior analysis of EASEL were categorized as new users of SGLT2i, even if they were eligible new users of SGLT2i and non‐SGLT2i at different times during the study, potentially introducing a lead‐in time bias.^31^ Therefore, we reanalysed the EASEL study to consider the potential for time‐varying exposure and allowed eligible patients to enter either respective arm of the study that corresponded to their active drug exposure (particularly for the on‐treatment period), decreasing the risk of time bias. We elected to focus on the specific SGLT2i canagliflozin and further differentiate CV from non‐CV causes of mortality.

## 2 MATERIALS AND METHODS

This was a retrospective new user cohort study using the Department of Defense (DoD) Military Health System (MHS) data, which integrates all medical, clinical, pharmacy and administrative data for every eligible MHS beneficiary across the United States. The DoD is composed of active or retired service members and their dependents, with approximately 10 million patients actively receiving care. In accordance with transparency and openness promotion guidelines, the analytic methods and study materials are stored at Health ResearchTx and could be made available to other researchers for purposes of reproducing the results or replicating the procedure.^10^

### 2.1 New users cohort creation

The study included 2 comparator cohorts: new users of canagliflozin or new users of non‐SGLT2i on top of standard‐of‐care therapy. The non‐SGLT2i cohort included dipeptidyl peptidase‐4 (DPP‐4) inhibitors, GLP‐1 receptor agonists, thiazolidinediones, sulfonylureas, insulin, and other AHAs (acarbose, bromocriptine, miglitol, nateglinide and repaglinide) and excluded metformin. Patients with any exposure to any other SGLT2i (ie, empagliflozin or dapagliflozin) were excluded. New users were defined as patients whose first exposure to a non‐metformin AHA during the study period from 4/1/2013 to 12/31/2016 occurred ≥365 days after the start of observation in the database, with no prior exposure to any medication within the same AHA medication class in the prior 365 days, and the date of the first dispensing of the therapy of interest was considered the index date. Eligible patients with T2DM were required to have ≥1 year of observation before the index date, with established CV disease (including coronary artery disease, heart failure, cerebrovascular disease and peripheral artery disease), and be ≥18 years of age. Patients with type 1 diabetes mellitus or secondary diabetes mellitus were excluded from this study. Patients were followed from the index date until the first occurrence of any of the following: (a) outcome of interest, (b) death, (c) disenrollment from the DoD or (d) last observation in the database.

The above analytical design was prespecified in the study protocol, noting that patients who met the new user criteria for both treatment arms were eligible for inclusion in both cohorts, as of the date of earliest initiation of each treatment, specifically addressing the study design issues raised by others.^31^, ^32^ For these patients, baseline characteristics were independently assessed as of each index date, and patients were available for potential matching in both instances to an eligible subject from the other treatment arm. Patients who initiated canagliflozin and a non‐SGLT2i on the same day were excluded from the analysis.

Exposure propensity score (EPS) matching was used to reduce confounding due to imbalance in baseline covariates. A regularized logistic regression model was used to estimate the predicted probability of patients receiving canagliflozin, and canagliflozin new users were EPS‐matched to new users of non‐SGLT2i in a 1:1 ratio. Approximately 1000 variables were considered for inclusion in the model, including patient demographics and characteristics, duration of diabetes, baseline comorbidities and medication use, comprehensive diagnoses and procedures mapped to respective Clinical Classifications Software categories, a calculated Charlson Comorbidity Index (CCI) score and various healthcare resource utilization measures. No missing data imputation methods were applied in any calculation of prevalence rates for baseline covariates or incidence rates for the outcomes of interest. If a medical condition was not observed in the patient's record, then this condition was assumed not present. Baseline measures were assessed over 2 periods, the full pre‐index period spanning back to 1 April 2008, and a 1‐year pre‐index period, with the ability for all variables across both periods to be included in the final model. The number of unique baseline AHA medications was included in the EPS model to factor in differences in background AHA therapy. By design, the new use of other non‐SGLT2i defined the control group and necessitated specific prescriptions of these drugs before the index date not to be included in the EPS estimation to avoid multicollinearity. Procedure and diagnostic codes used to identify comorbidities have been validated in previous studies.^3^, ^4^, ^6^, ^9^, ^11^, ^12^, ^13^, ^16^, ^19^, ^20^, ^21^, ^27^, ^33^, ^34^, ^35^ Additional details of EPS matching have been published.^38^

### 2.2 Study outcomes

The primary outcome of the study was the composite of ACM and HHF. In addition, a composite of MACE (CV death, nonfatal MI and nonfatal stroke), an expanded MACE outcome that included HHF, a modified MACE that included non‐CV death (ACM, nonfatal MI and nonfatal stroke), and a composite of modified MACE + HHF, as well as the individual components of the composite end‐points, were evaluated. MI and stroke events were considered nonfatal if patients did not die during hospitalization for the index event. BKLE amputation was assessed as a safety end‐point and includes both minor (digits, partial foot and ankle disarticulation) and major (below‐knee) amputations.

ACM was defined as any record of death regardless of cause. To differentiate the cause of death, patients who died were linked with the National Death Index (NDI), which utilizes coroner records and other available sources to determine cause of death.^22^ CV death was defined using the standard recommended by the American Heart Association (*International Classification of Diseases, Tenth Revision* [*ICD‐10*] diagnostic codes for diseases of the circulatory system [I00‐I99] and congenital malformations of the circulatory system [Q20‐Q28]).^2^ MI, stroke and HHF were ascertained based on *International Classification of Diseases, Ninth Revision* (*ICD‐9*) and *ICD‐10* diagnosis codes, and BKLE amputation was ascertained based on *ICD‐9* and *ICD‐10* procedure codes, consistent with our prior work (Table S1).^38^ Patients with a history of BKLE amputation events before the index exposure were excluded from comparative analyses of BKLE amputation to avoid confounding due to inherent intrasubject risk, potential for reverse causation and potential for immortal time bias in the situation in which such patients may no longer be at risk for future BKLE amputation events at the location of a prior amputation.

### 2.3 Statistical analysis

The statistical methods employed in this study were consistent with those described previously.^38^ Specifically, conditional Cox proportional hazards regression based on time to first event was used to estimate hazard ratios (HRs) and 95% confidence intervals (CIs), comparing the treatment effect of canagliflozin against non‐SGLT2i (reference group) in relation to each study end‐point using both intent‐to‐treat (ITT) and on‐treatment approaches. For the ITT analysis, time at risk was calculated from the index date until the occurrence of an outcome of interest or the end of observation, whichever occurred first. It is worth noting for the on‐treatment analyses of patients in both cohorts that follow‐up was censored for one arm at the time of crossover from or to SGLT2i exposure unless follow‐up time had already been censored for another reason described above. Kaplan‐Meier plots were generated to characterize the contour of risk over time for each outcome. Because the results were generally consistent between both approaches, for the purpose of this reporting, we primarily focused on the ITT results, unless otherwise specified. Although the formal statistical analyses focused on the comparison of canagliflozin new users versus non‐SGLT2i new users, additional descriptive data (eg, event rates) were summarized based on individual non‐SGLT2i therapeutic classes (ie, DPP‐4 inhibitors, GLP‐1 receptor agonists, thiazolidinediones, sulfonylureas, insulin and other AHAs).

Due to the potential heterogeneity of non‐SGLT2i new users (eg, insulin use may represent an advanced stage of T2DM and sulfonylureas may be associated with heart failure‐related outcomes), sensitivity analyses were conducted to assess whether the study findings were driven by any particular subset of patients. As part of sensitivity analyses, patients receiving insulin, sulfonylureas and thiazolidinediones were removed (individually and collectively) from the non‐SGLT2i cohort along with their canagliflozin matching pairs to further evaluate treatment effect, as done previously.^7^, ^14^ Several subgroup analyses were prespecified, including sex, age, insulin use, GLP‐1 receptor agonist use, history of heart failure, recent HHF (past 12 months), number of CV risk factors (ie, CV disease, coronary artery disease, peripheral vascular disease), renal disease by CCI score and chronic renal disease.

The study protocol was reviewed and approved by the DoD Institutional Review Board, and all analyses were performed by a research organization^10^ using SAS V9.4 (SAS Institute Inc).

## 3 RESULTS

### 3.1 Study population

Overall, 7713 new users of canagliflozin and 102 516 new users of a non‐SGLT2i with T2DM and established CV disease were identified during the study period (Figure S1). There were 99 (1.3%) patients who started canagliflozin and a non‐SGLT2i on the same day and were excluded. After EPS matching, 7697 (99.8% of the total eligible) new users of canagliflozin were matched 1:1 with 7697 new users of a non‐SGLT2i, for a total of 15 394 patients. In this PS‐matched cohort, 888 new users were eligible for and assigned to both cohorts (ie, 521 were new users of non‐SGLT2i before canagliflozin and 367 were new users of non‐SGLT2i after canagliflozin).

Key clinical characteristics among new users of canagliflozin and non‐SGLT2i before and after matching are shown in Table 1. Before matching, patients in the canagliflozin cohort were younger and had longer durations of T2DM compared with the non‐SGLT2i cohort. By design and as expected, the canagliflozin cohort had greater usage of baseline AHA medications compared with the non‐SGLT2i cohort, and differences in non‐AHA medication use were observed between cohorts. Differences were also observed among most comorbidities of interest, with the canagliflozin cohort having lower prevalence of many baseline comorbidities, as well as lower CCI scores, compared to the non‐SGLT2i cohort.

**Table 1:** Baseline characteristics by treatment cohort before and after propensity matching^a^

Characteristic	Before matching		After matching	
	Canagliflozin (n = 7713)	Non‐SGLT2i (n = 102 516)	Canagliflozin (n = 7697)	Non‐SGLT2i (n = 7697)
Age, year^b^	65.6 (8.9)	69.4 (10.5)	65.6 (8.9)	65.7 (9.7)
Sex, %				
Male	56.1	56.2	56.2	55.3
Female	43.9	43.8	43.8	44.7
Race, %				
White	35.8	27.2	35.8	33.9
Black	5.3	6.0	5.3	5.9
Asian or Pacific Islander	1.6	1.7	1.6	1.7
Other^c^	57.4	65.1	57.4	58.5
T2DM duration, y^b^	5.4 (1.8)	5.0 (2.2)	5.4 (1.8)	5.5 (1.9)
CV disease duration, y^b^	4.2 (2.1)	4.3 (2.2)	4.2 (2.1)	4.2 (2.1)
Charlson Comorbidity Index score^b^	4.9 (2.4)	6.0 (3.1)	4.9 (2.4)	4.9 (2.5)
Comorbidities of interest, %				
Atrial fibrillation	9.0	14.8	9.0	9.2
AIDS/HIV	0.1	0.1	0.1	0.0
Cardiomyopathy	3.9	6.4	3.9	3.6
Cerebrovascular disease	14.5	19.9	14.5	14.9
Congestive heart failure	10.5	18.8	10.5	10.2
Chronic obstructive pulmonary disease	21.8	27.8	21.8	22.7
Dementia	0.9	3.6	0.9	1.5
Diabetes mellitus with chronic complications^d^	31.3	29.8	31.2	29.6
Hemiplegia/paraplegia	0.6	1.7	0.6	0.6
Hepatic disease	7.9	7.4	7.9	7.5
Hyperlipidemia	75.3	70.8	75.3	75.5
Hypertension	86.7	86.0	86.6	85.7
Ischaemic stroke	3.6	6.7	3.6	3.8
Malignancy	9.2	12.9	9.2	9.0
Mild liver disease	7.8	7.3	7.8	7.4
Moderate/severe liver disease	0.5	0.7	0.5	0.4
MI	5.8	8.8	5.8	5.6
Peptic ulcer disease	1.1	1.5	1.1	1.0
Peripheral vascular disease	15.4	20.2	15.4	15.5
Renal disease	10.6	21.4	10.7	11.4
Rheumatic disease	2.7	3.7	2.7	3.2
Metastatic solid tumour	0.5	1.8	0.5	0.8
Transient ischaemic attack	3.1	3.9	3.1	3.0
Venous thromboembolism	2.4	4.3	2.4	2.8
Medications of interest, %				
ACE inhibitor	41.6	40.5	41.7	41.0
ARB	37.4	31.3	37.4	37.5
ACE inhibitor and/or ARB	75.2	68.1	75.2	74.6
Antiarrhythmics	2.2	3.4	2.2	2.1
β‐blockers	49.7	51.8	49.7	50.4
Calcium channel blockers	5.9	6.6	5.9	5.5
Digoxin	3.2	4.1	3.2	2.5
Non‐loop diuretics	18.5	19.7	18.5	19.2
Loop diuretics	17.7	23.0	17.7	18.2
Statins or ezetimibe	82.1	73.9	82.1	81.7
NSAIDs	45.9	44.0	45.9	45.9
Anticoagulants	9.3	12.8	9.3	8.8
Number of AHA medications^b^	2.8 (1.5)	1.4 (1.2)	2.8 (1.5)	2.8 (1.5)
AHA therapies^e^				
Insulin	26.4	7.0	26.3	18.1
Metformin (any)	78.9	63.4	79.0	83.4
Sulfonylurea	47.9	24.7	47.8	48.5
Thiazolidinediones	13.5	6.2	13.5	14.4
GLP‐1 receptor agonists	22.4	3.0	22.3	10.5
DPP‐4 inhibitors	59.5	16.3	59.4	35.5
Metformin plus ≥1 AHA	71.8	30.1	71.8	64.6
Other	3.6	1.2	3.6	3.2

Abbreviations: ACE, angiotensin‐converting enzyme; AHA, antihyperglycemic agent; AIDS/HIV, acquired immunodeficiency syndrome/human immunodeficiency virus; ARB, angiotensin receptor blocker; CV, cardiovascular; DPP‐4, dipeptidyl peptidase‐4; EPS, exposure propensity score; GLP‐1, glucagon‐like peptide‐1; MI, myocardial infarction; NSAID, nonsteroidal anti‐inflammatory drug; SD, standard deviation; SGLT2i, sodium glucose co‐transporter 2 inhibitor; T2DM, type 2 diabetes mellitus.

a Between‐cohort standardized difference <0.1 for all covariates listed.

b Data are mean (SD).

c Includes American Indian or Alaskan Native, other and unknown/missing.

d As defined by Charlson Comorbidity Index score.

e Individual AHA therapies were not included in EPS matching and are presented for descriptive purposes. Therefore, standardized differences may not meet the <0.1 threshold after matching.

After EPS matching, all baseline patient characteristics included in the EPS model were well balanced (standardized differences <0.1 for all baseline characteristics after propensity matching; Figure S2). Among the matched cohort, the mean age was 65.6 (standard deviation [SD], 9.3) years, 44.3% were female, the mean duration of T2DM was 5.4 (SD, 1.8) years, and the mean duration of CV disease was 4.2 (SD, 2.1) years. Histories of hypertension (86.2%), hyperlipidemia (75.4%), chronic obstructive pulmonary disease (22.3%), peripheral vascular disease (15.5%) and cerebrovascular disease (14.7%) were fairly prevalent, and 81.2% of patients were treated with metformin and 22.2% with insulin at baseline.

The median ITT follow‐up time was 2.03 years (interquartile range, 1.29, 2.82), which was similar between cohorts (2.00 and 2.08 years with canagliflozin and non‐SGLT2i, respectively). The median on‐treatment follow‐up time was 0.71 (interquartile range, 0.25, 1.49; 0.68; and 0.74 years with canagliflozin and non‐SGLT2i, respectively).

Among the 555 patients with an ACM outcome in this analysis, the cause of death for 552 (99.5%) patients was ascertained based on the NDI file. The remaining 3 patients, for whom a cause of death could not be determined, were excluded, along with their respective match, from comparative analyses that included CV and non‐CV death.

### 3.2 CV and mortality outcomes

**Figure 1:**
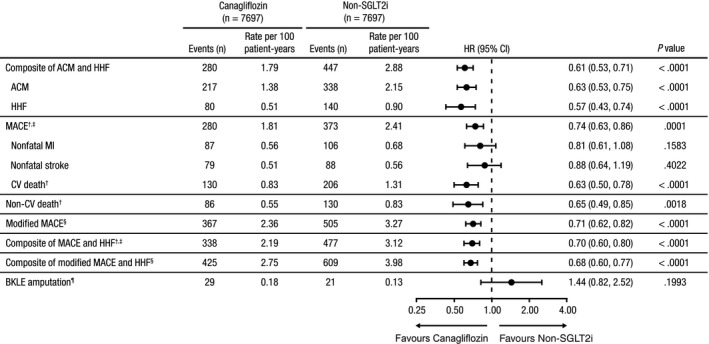
Risk of CV, mortality and BKLE amputation outcomes for patients in the propensity‐matched ITT cohort. ACM, all‐cause mortality; BKLE, below‐knee lower extremity; CI, confidence interval; CV, cardiovascular; HHF, hospitalization for heart failure; HR, hazard ratio; ITT, intent‐to‐treat; MACE, major adverse cardiovascular events; MI, myocardial infarction; NDI, National Death Index; SGLT2i, sodium glucose co‐transporter 2 inhibitor. ^†^Patients with an ACM outcome without NDI data (n = 3) were removed from analyses along with their matched pair. ^‡^MACE is the composite of CV death, nonfatal MI and nonfatal stroke. ^§^Modified MACE is the composite of ACM, nonfatal MI and nonfatal stroke. ^¶^Patients with prior BKLE amputation (n = 6) were removed from analyses along with their matched pair

**Figure 2:**
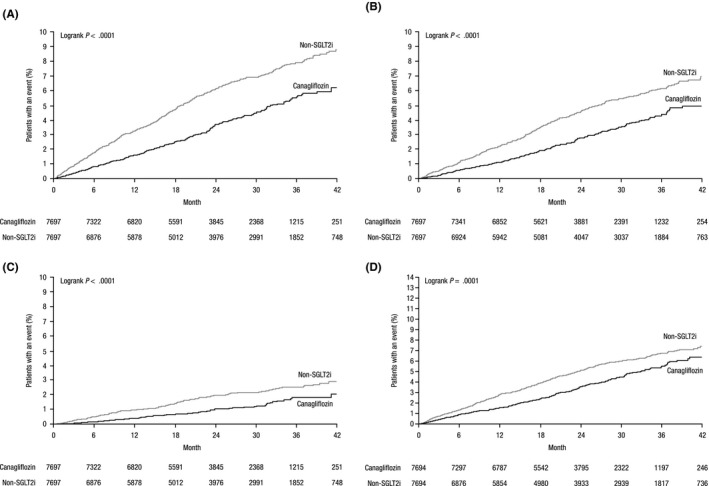
Event curves for (A) the primary composite outcome, (B) ACM, (C) HHF and (D) MACE in the propensity‐matched ITT cohort.^†^ ACM, all‐cause mortality; HHF, hospitalization for heart failure; ITT, intent‐to‐treat; MACE, major adverse cardiovascular event; SGLT2i, sodium glucose co‐transporter 2 inhibitor. ^†^Propensity‐matched using an exposure propensity score

The primary composite outcome of ACM and HHF and secondary CV outcomes for patients in the EPS‐matched ITT cohort are shown in Figure 1. The incidence rate of the primary outcome was 1.79 versus 2.88 per 100 person‐years among new users of canagliflozin and non‐SGLT2i, respectively (HR, 0.61; 95% CI, 0.53‐0.71; *P* < .0001; Figure 1). Similarly, initiation of canagliflozin was associated with a lower rate of ACM (1.38 vs 2.15 per 100 person‐years; HR, 0.63; 95% CI, 0.53‐0.75; *P* < .0001) and HHF (0.51 vs 0.90 per 100 person‐years; HR, 0.57; 95% CI, 0.43‐0.74; *P* < .0001) compared with non‐SGLT2i. For these outcomes, the treatment benefit associated with canagliflozin started early and persisted over the study period (Figure 2).

Based on the NDI cause of death data, CV death was analysed as part of a composite outcome of MACE (CV death, nonfatal MI and nonfatal stroke). The rate of MACE was lower in new users of canagliflozin compared with new users of non‐SGLT2i (1.81 vs 2.41 per 100 patient‐years; HR, 0.74; 95% CI, 0.63‐0.86; *P* = .0001). The rates of the individual MACE components of nonfatal MI (0.56 vs 0.68 per 100 patient‐years; HR, 0.81; 95% CI, 0.61‐1.08; *P* = .16) and nonfatal stroke (0.51 vs 0.56 per 100 patient‐years; HR, 0.88; 95% CI, 0.64‐1.19; *P* = .40) were not significantly different. The rate of CV death was higher than developing either nonfatal atherosclerotic event in each treatment group. The rate of CV death was lower in new users of canagliflozin compared with new users of non‐SGLT2i (0.83 vs 1.31 per 100 patient‐years; HR, 0.63; 95% CI, 0.50‐0.78; *P* < .0001), and similar reductions were seen for non‐CV death (0.55 vs 0.83 per 100 patient‐years; HR, 0.65; 95% CI, 0.49‐0.85; *P* = .002) compared with non‐SGLT2i. In addition, the rate of a modified MACE outcome substituting ACM for CV death (ie, ACM, nonfatal MI and nonfatal stroke) was lower in new users of canagliflozin compared with new users of non‐SGLT2i (2.36 vs 3.27 per 100 patient‐years; HR, 0.71; 95% CI, 0.62‐0.82; *P* < .0001). Furthermore, the rate of the composite of MACE and HHF was significantly lower among new users of canagliflozin compared with new users of a non‐SGLT2i (2.19 vs 3.12 per 100 patient‐years; HR, 0.70; 95% CI, 0.60‐0.80; *P* < .0001). Consistent results were observed in a composite of modified MACE and HHF (2.75 vs 3.98 per 100 patient‐years with canagliflozin and non‐SGLT2i, respectively; HR, 0.68; 95% CI, 0.60‐0.77; *P* < .0001). In the on‐treatment analyses, lower event rates were generally seen among active canagliflozin patients (Figure S3).

Analysis of the primary outcome in prespecified subgroups showed a consistent benefit of canagliflozin treatment compared with non‐SGLT2i among each of the subgroups based on sex, age, insulin or GLP‐1 receptor agonist use in the past 12 months, history of heart failure, HHF in the past 12 months, number of cerebrovascular risk factors and renal disease, with no between‐subgroup heterogeneity detected (Figure S4). Results of sensitivity analyses that removed patients treated with insulin, sulfonylureas and thiazolidinediones at baseline, individually and in combination, were generally quantitatively consistent with the overall study results, suggesting that none of these medications were disproportionally impacting the final results (Figure S5).

### 3.3 Safety outcome

Excluding patients with previous BKLE amputation events (n = 6) and their respective matches, a total of 50 new BKLE amputation events were observed in the ITT cohort and 14 events in the on‐treatment cohort. In the ITT analysis, the incidence rate of BKLE amputation was not significantly different, with 29 and 21 events in new users of canagliflozin and non‐SGLT2i, respectively (0.18 vs 0.13 per 100 person‐years; HR, 1.44; 95% CI, 0.82‐2.52; *P* = .20; Figure 1). Similar results were observed in the on‐treatment analysis with 7 BKLE amputation events in each cohort (0.10 vs 0.08 per 100 person‐years with canagliflozin and non‐SGLT2i, respectively; HR, 1.26; 95% CI, 0.44‐3.55; *P* = .67; Figure S3). Because the number of events was relatively limited, the CI for the HR is quite wide and contains the point estimate that was observed in the CANVAS Program.^23^ Generally consistent results were observed among all prespecified subgroups (Figure S6).

## 4 DISCUSSION

EASEL is a collaborative population‐based study of patients with T2DM and established CV disease enrolled in one of the largest public health insurance claims databases in the United States. In the present analysis, we examined the clinical effectiveness and safety of canagliflozin in routine clinical practice using a study design that minimized the risk of potential selection bias. We also linked data with the NDI to ascertain CV death and focused our analyses on new users of canagliflozin. Compared with patients initiated on non‐SGLT2i, patients initiated on canagliflozin had a significantly lower risk of ACM, HHF, MACE and CV and non‐CV related causes of death. Incorporation of the lead‐in time and better accounting for follow‐up time from additional non‐SGLT2i exposure had a limited impact on the overall study findings.^38^ The lower risk of MACE and HHF observed with initiation of canagliflozin and numerically higher rate of BKLE amputation is consistent with the results of the CANVAS Program and other observational studies of SGLT2i to date.^23^, ^25^, ^28^, ^39^ There are multiple ongoing large CV outcome trials studying SGLT2i in patients with T2DM with and without established CV disease, as well as in patients with chronic kidney disease and heart failure with and without T2DM.^17^, ^30^ Moreover, there are multiple ongoing population‐based studies investigating the effectiveness and safety of these drugs in less selected patients. The results of these studies are crucial to further understanding the benefits and safety of SGLT2i, including canagliflozin, in broad populations being recommended for treatment that may differ from the strict selection of trial participants.^36^, ^37^

The ITT and on‐treatment analyses resulted in fairly consistent results, with an attenuation in the effect sizes of mortality and other CV outcomes in the ITT cohort, particularly in HHF and non‐CV death. Attenuation of effects in the ITT cohort is likely due to a median 1.32 years of additional follow‐up time when patients were not taking the intended treatment, during which time there would be no meaningful treatment effect. Reciprocally, the observation is also consistent with a presumed hemodynamic effect rather than glycemic or metabolic disease modifying effects of SGLT2i. The effects were relatively consistent across patient subgroups and in sensitivity analyses excluding patients on insulin, sulfonylureas and thiazolidinediones. Less is known about the time‐relationship between SGLT2i treatment and BKLE amputation risk, and the limited number of amputation events restricts us from further conjecture.^23^

This analysis has several strengths. First, with >110 000 new users of canagliflozin or non‐SGLT2i, comparable treatment cohorts were established though EPS matching that preserved >99% of eligible canagliflozin new users. The DoD MHS database is representative of many of the demographic and clinical characteristics of the US population and generally has longer longitudinal follow‐up than other commercial databases. Additionally, NDI records were used to identify CV and non‐CV related death among >99% of patients with fatal events. The study design also minimized, to the extent possible, the risk of lead‐in time bias, a concern for observational studies.^31^ Lead‐in time bias is the result of excluding or misclassifying the time at risk (both lead‐in and follow‐up time) when patients are treated with the exposure or comparator drug, which may exaggerate observed benefits. Furthermore, in the on‐treatment analysis, follow‐up time was censored when a non‐SGLT2i was initiated in patients in the canagliflozin cohort or when canagliflozin was initiated in patients in the non‐SGLT2i cohort, and these results corroborated the ITT analyses.

However, there are limitations common to observational studies to note, which include the potential for unmeasured confounding and residual bias. Extensive propensity matching was used to reduce this risk; however, clinical variables, such as HbA1c, estimated glomerular filtration rate, body mass index, blood pressure and microvascular complications of diabetes, were not used for propensity score matching as they were not available in the database. Additionally, residual imbalance remained in the use of different classes of AHA medications, which were not included in the propensity matching algorithm and may represent selection bias for new use of an SGLT2i over a non‐SGLT2i. Nevertheless, clinically, this may be analogous to clinical trial designs in which interventional therapies are added on top of standard‐of‐care therapy. Additionally, pharmacologic dispensing records were used to infer medication use, but dispensing records do not ensure that drugs were taken as prescribed. On average, the observational time during the follow‐up period was shorter compared with the CANVAS Program. Finally, the population included in the DoD database may be different in terms of demographics and healthcare access compared with the general population of the United States; therefore, the results may not be generalizable.

In conclusion, this subsequent analysis of the EASEL study showed that in patients with T2DM and high CV risk treated in routine clinical practice in a large US DoD healthcare system, initiation of canagliflozin treatment was associated with a lower risk of CV events, CV death and ACM, with no significant increase in the risk of BKLE amputation compared with non‐SGLT2i treatment, though statistical power was limited because of the limited number of events for this safety end‐point.

## CONFLICT OF INTEREST

JAU has received consulting fees from Amgen, Boehringer Ingelheim, Janssen Research & Development, LLC, Merck, Novartis and Sanofi Pasteur; speaking honoraria from Boehringer Ingelheim and Janssen; and research grant support from AstraZeneca and Novartis. ZY, PR and NR are full‐time employees of Janssen Research & Development, LLC. TR and NMS are full‐time employees of Health ResearchTx, LLC, which has a business relationship with Janssen. MG reports no conflicts of interest.

## AUTHOR CONTRIBUTIONS

JAU, ZY, PR, TR, NMS, MG and NR were involved in the design of the study, collection of data or analysis of data and preparation of the final manuscript.

## ETHICS STATEMENT

The study protocol was reviewed and approved by the DoD Institutional Review Board.

## Data Availability

In accordance with transparency and openness promotion guidelines, the analytic methods and study materials are stored at Health ResearchTx and could be made available to other researchers for purposes of reproducing the results or replicating the procedure.
