# Supplementary material for: Cardiovascular outcomes and mortality after initiation of canagliflozin: Analyses from the EASEL Study
Source: Endocrinol Diabetes Metab. 2019 Oct 15;3(1):e00096. doi: 10.1002/edm2.96 (PMC6947703; doi:10.1002/edm2.96)
Supplement: Supplementary file 1 [file EDM2-3-e00096-s001.docx]

**SUPPLEMENTAL MATERIALS**

**Online Table 1. Procedure and Diagnosis Codes for HHF, Stroke, MI, and BKLE Amputation**

| **Concept code** | **Concept name** | **Vocabulary** |
| --- | --- | --- |
| **HHF** |  |  |
| 428.x | Heart failure | *ICD-9* |
| I50.x | Heart failure | *ICD-10* |
| **Stroke** |  |  |
| 433.x1 | Occlusion and stenosis of precerebral arteries- carotid artery | *ICD-9* |
| 434.x1 | Occlusion of cerebral arteries- cerebral embolism | *ICD-9* |
| 436 | Acute but ill-defined cerebrovascular disease | *ICD-9* |
| I63.x | Cerebral infarction | *ICD-10* |
| I67.8x | Other specified cerebrovascular disease | *ICD-10* |
| 430 | Subarachnoid hemorrhage | *ICD-9* |
| 431 | Intracerebral hemorrhage | *ICD-9* |
| I60.x | Nontraumatic subarachnoid hemorrhage | *ICD-10* |
| I61.x | Nontraumatic intracerebral hemorrhage | *ICD-10* |
| **MI** |  |  |
| 410.x | Acute myocardial infarction | *ICD-9* |
| I21.x | ST elevation (STEMI) and non-ST elevation (NSTEMI) myocardial infarction | *ICD-10* |
|  |  |  |
| **BKLE Amputation** | |  |
| 27880 | Amputation, leg, through tibia and fibula | *CPT-4* |
| 27881 | Amputation, leg, through tibia and fibula; with immediate fitting technique including application of first cast | *CPT-4* |
| 27882 | Amputation, leg, through tibia and fibula; open, circular (guillotine) | *CPT-4* |
| 27886 | Amputation, leg, through tibia and fibula; re-amputation | *CPT-4* |
| 27888 | Amputation, ankle, through malleoli of tibia and fibula (eg, Syme, Pirogoff type procedures), with plastic closure and resection of nerves | *CPT-4* |
| 28800 | Amputation, foot; midtarsal (eg, Chopart type procedure) | *CPT-4* |
| 28805 | Amputation, foot; transmetatarsal | *CPT-4* |
| 28810 | Amputation, metatarsal, with toe, single | *CPT-4* |
| 28820 | Amputation, toe; metatarsophalangeal joint | *CPT-4* |
| 28825 | Amputation, toe; interphalangeal joint | *CPT-4* |
| 84.11 | Amputation of toe | *ICD-9* |
| 0Y6P0Z0 | Detachment at right 1st toe, complete, open approach | *ICD-10* |
| 0Y6P0Z1 | Detachment at right 1st toe, high, open approach | *ICD-10* |
| 0Y6P0Z2 | Detachment at right 1st toe, mid, open approach | *ICD-10* |
| 0Y6P0Z3 | Detachment at right 1st toe, low, open approach | *ICD-10* |
| 0Y6Q0Z0 | Detachment at left 1st toe, complete, open approach | *ICD-10* |
| 0Y6Q0Z1 | Detachment at left 1st toe, high, open approach | *ICD-10* |
| 0Y6Q0Z2 | Detachment at left 1st toe, mid, open approach | *ICD-10* |
| 0Y6Q0Z3 | Detachment at left 1st toe, low, open approach | *ICD-10* |
| 0Y6R0Z0 | Detachment at right 2nd toe, complete, open approach | *ICD-10* |
| 0Y6R0Z1 | Detachment at right 2nd toe, high, open approach | *ICD-10* |
| 0Y6R0Z2 | Detachment at right 2nd toe, mid, open approach | *ICD-10* |
| 0Y6R0Z3 | Detachment at right 2nd toe, low, open approach | *ICD-10* |
| 0Y6S0Z0 | Detachment at left 2nd toe, complete, open approach | *ICD-10* |
| 0Y6S0Z1 | Detachment at left 2nd toe, high, open approach | *ICD-10* |
| 0Y6S0Z2 | Detachment at left 2nd toe, mid, open approach | *ICD-10* |
| 0Y6S0Z3 | Detachment at left 2nd toe, low, open approach | *ICD-10* |
| 0Y6T0Z0 | Detachment at right 3rd toe, complete, open approach | *ICD-10* |
| 0Y6T0Z1 | Detachment at right 3rd toe, high, open approach | *ICD-10* |
| 0Y6T0Z2 | Detachment at right 3rd toe, mid, open approach | *ICD-10* |
| 0Y6T0Z3 | Detachment at right 3rd toe, low, open approach | *ICD-10* |
| 0Y6U0Z0 | Detachment at left 3rd toe, complete, open approach | *ICD-10* |
| 0Y6U0Z1 | Detachment at left 3rd toe, high, open approach | *ICD-10* |
| 0Y6U0Z2 | Detachment at left 3rd toe, mid, open approach | *ICD-10* |
| 0Y6U0Z3 | Detachment at left 3rd toe, low, open approach | *ICD-10* |
| 0Y6V0Z0 | Detachment at right 4th toe, complete, open approach | *ICD-10* |
| 0Y6V0Z1 | Detachment at right 4th toe, high, open approach | *ICD-10* |
| 0Y6V0Z2 | Detachment at right 4th toe, mid, open approach | *ICD-10* |
| 0Y6V0Z3 | Detachment at right 4th toe, low, open approach | *ICD-10* |
| 0Y6W0Z0 | Detachment at left 4th toe, complete, open approach | *ICD-10* |
| 0Y6W0Z1 | Detachment at left 4th toe, high, open approach | *ICD-10* |
| 0Y6W0Z2 | Detachment at left 4th toe, mid, open approach | *ICD-10* |
| 0Y6W0Z3 | Detachment at left 4th toe, low, open approach | *ICD-10* |
| 0Y6X0Z0 | Detachment at right 5th toe, complete, open approach | *ICD-10* |
| 0Y6X0Z1 | Detachment at right 5th toe, high, open approach | *ICD-10* |
| 0Y6X0Z2 | Detachment at right 5th toe, mid, open approach | *ICD-10* |
| 0Y6X0Z3 | Detachment at right 5th toe, low, open approach | *ICD-10* |
| 0Y6Y0Z0 | Detachment at left 5th toe, complete, open approach | *ICD-10* |
| 0Y6Y0Z1 | Detachment at left 5th toe, high, open approach | *ICD-10* |
| 0Y6Y0Z2 | Detachment at left 5th toe, mid, open approach | *ICD-10* |
| 0Y6Y0Z3 | Detachment at left 5th toe, low, open approach | *ICD-10* |
| 84.12 | Amputation through foot | *ICD-9* |
| 0Y6M0Z4 | Detachment at right foot, complete 1st ray, open approach | *ICD-10* |
| 0Y6M0Z5 | Detachment at right foot, complete 2nd ray, open approach | *ICD-10* |
| 0Y6M0Z6 | Detachment at right foot, complete 3rd ray, open approach | *ICD-10* |
| 0Y6M0Z7 | Detachment at right foot, complete 4th ray, open approach | *ICD-10* |
| 0Y6M0Z8 | Detachment at right foot, complete 5th ray, open approach | *ICD-10* |
| 0Y6M0Z9 | Detachment at right foot, partial 1st ray, open approach | *ICD-10* |
| 0Y6M0ZB | Detachment at right foot, partial 2nd ray, open approach | *ICD-10* |
| 0Y6M0ZC | Detachment at right foot, partial 3rd ray, open approach | *ICD-10* |
| 0Y6M0ZD | Detachment at right foot, partial 4th ray, open approach | *ICD-10* |
| 0Y6M0ZF | Detachment at right foot, partial 5th ray, open approach | *ICD-10* |
| 0Y6N0Z4 | Detachment at left foot, complete 1st ray, open approach | *ICD-10* |
| 0Y6N0Z5 | Detachment at left foot, complete 2nd ray, open approach | *ICD-10* |
| 0Y6N0Z6 | Detachment at left foot, complete 3nd ray, open approach | *ICD-10* |
| 0Y6N0Z7 | Detachment at left foot, complete 4th ray, open approach | *ICD-10* |
| 0Y6N0Z8 | Detachment at left foot, complete 5th ray, open approach | *ICD-10* |
| 0Y6N0Z9 | Detachment at left foot, partial 1st ray, open approach | *ICD-10* |
| 0Y6N0ZB | Detachment at left foot, partial 2nd ray, open approach | *ICD-10* |
| 0Y6N0ZC | Detachment at left foot, partial 3rd ray, open approach | *ICD-10* |
| 0Y6N0ZD | Detachment at left foot, partial 4th ray, open approach | *ICD-10* |
| 0Y6N0ZF | Detachment at left foot, partial 5th ray, open approach | *ICD-10* |
| 84.13 | Disarticulation of ankle | *ICD-9* |
| 0Y6M0Z0 | Detachment at right foot, complete, open approach | *ICD-10* |
| 0Y6N0Z0 | Detachment at left foot, complete, open approach | *ICD-10* |
| 84.14 | Amputation of ankle through malleoli of tibia and fibula | *ICD-9* |
| 0Y6H0Z3 | Detachment at right lower leg, low, open approach | *ICD-10* |
| 0Y6J0Z3 | Detachment at left lower leg, low, open approach | *ICD-10* |
| 84.15 | Other amputation below knee | *ICD-9* |
| 0Y6H0Z1 | Detachment at right lower leg, high, open approach | *ICD-10* |
| 0Y6H0Z2 | Detachment at right lower leg, mid, open approach | *ICD-10* |
| 0Y6H0Z3 | Detachment at right lower leg, low, open approach | *ICD-10* |
| 0Y6J0Z1 | Detachment at left lower leg, high, open approach | *ICD-10* |
| 0Y6J0Z2 | Detachment at left lower leg, mid, open approach | *ICD-10* |
| 0Y6J0Z3 | Detachment at left lower leg, low, open approach | *ICD-10* |
| 84.16 | Disarticulation of knee | *ICD-9* |
| 0Y6F0ZZ | Detachment at right knee region, open approach | *ICD-10* |
| 0Y6G0ZZ | Detachment at left knee region, open approach | *ICD-10* |

BKLE, below-knee lower extremity; *CPT-4*, *Current Procedural Terminology, 4th Edition*; HHF, hospitalization for heart failure; *ICD-9*, *International Classification of Disease, Ninth Revision*; *ICD-10*, *International Classification of Disease, Tenth Revision*; MI, myocardial infarction.

**Online Figure 1. Patient attrition diagram.**

**
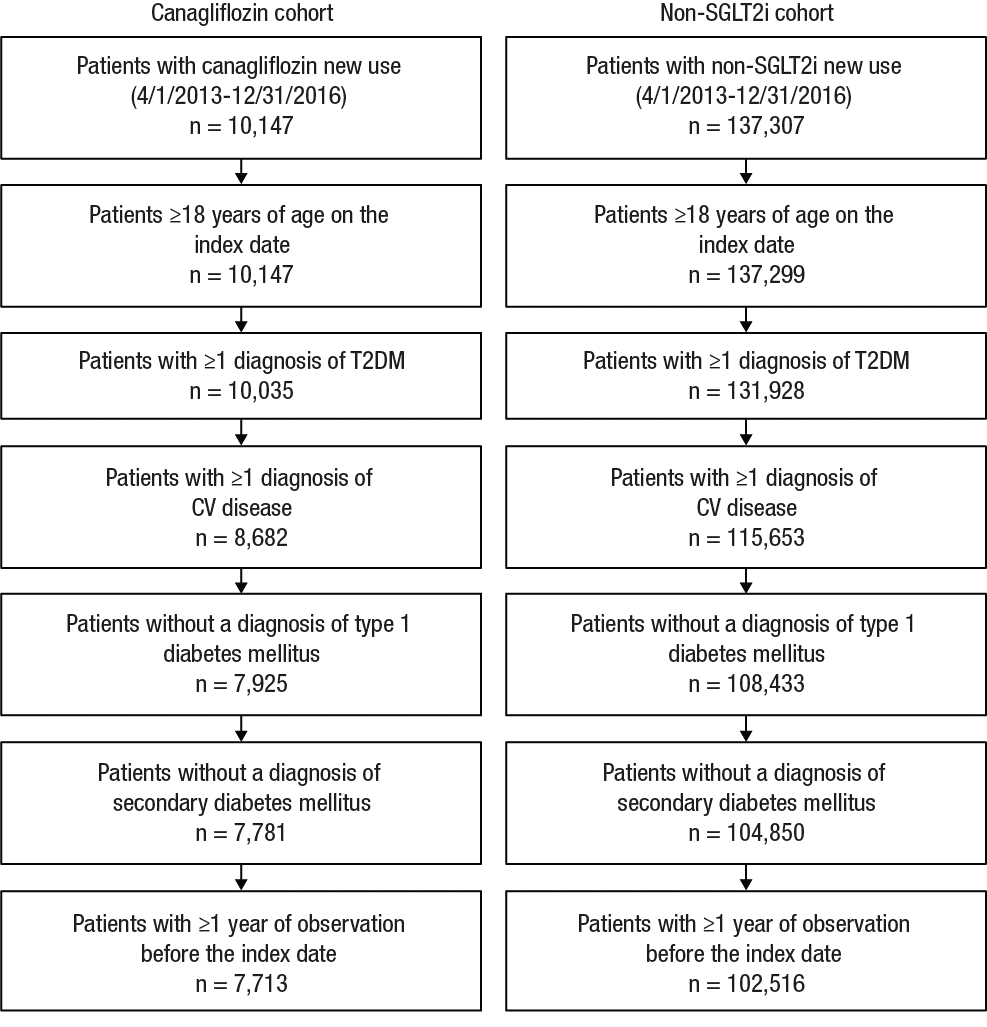
**

CV, cardiovascular; SGLT2i, sodium glucose co-transporter 2 inhibitor; T2DM, type 2 diabetes mellitus.

**Online Figure 2. Covariate balance of total baseline characteristics.**

**Online Figure 3. Risk of CV, mortality, and BKLE amputation outcomes for patients in the propensity-matched on-treatment cohort.**

**
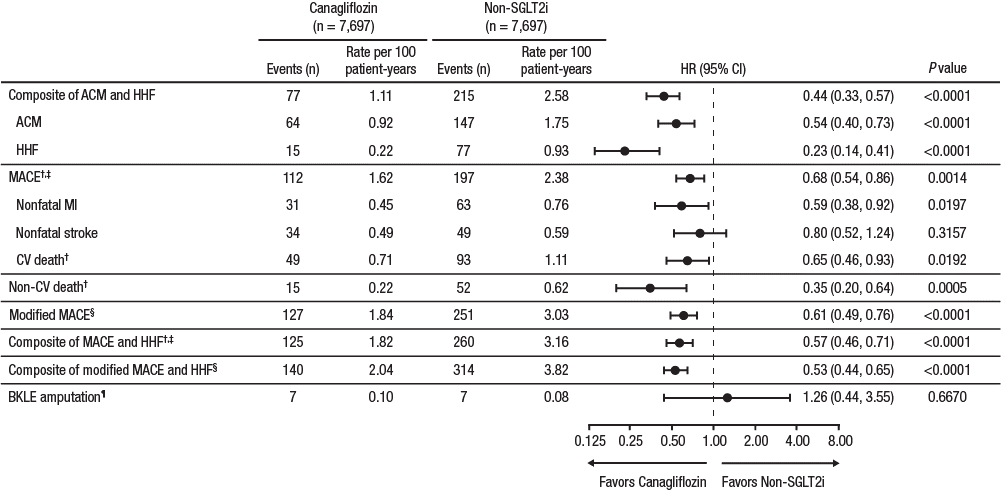
**

ACM, all-cause mortality; BKLE, below-knee lower extremity; CI, confidence interval; CV, cardiovascular; HHF, hospitalization for heart failure; HR, hazard ratio; MACE, major adverse cardiovascular events; MI, myocardial infarction; NDI, National Death Index; SGLT2i, sodium glucose co-transporter 2 inhibitor.

^†^Patients with an ACM outcome without NDI data (n = 3) were removed from analyses along with matched pair.

^‡^MACE is the composite of CV death, nonfatal MI, and nonfatal stroke.

^§^Modified MACE is the composite of ACM, nonfatal MI, and nonfatal stroke.

^¶^Patients with prior BKLE amputation (n = 6) were removed from analyses along with matched pair.

**Online Figure 4. Risk of the primary outcome in the propensity-matched ITT cohort stratified by treatment status and baseline subgroups.**


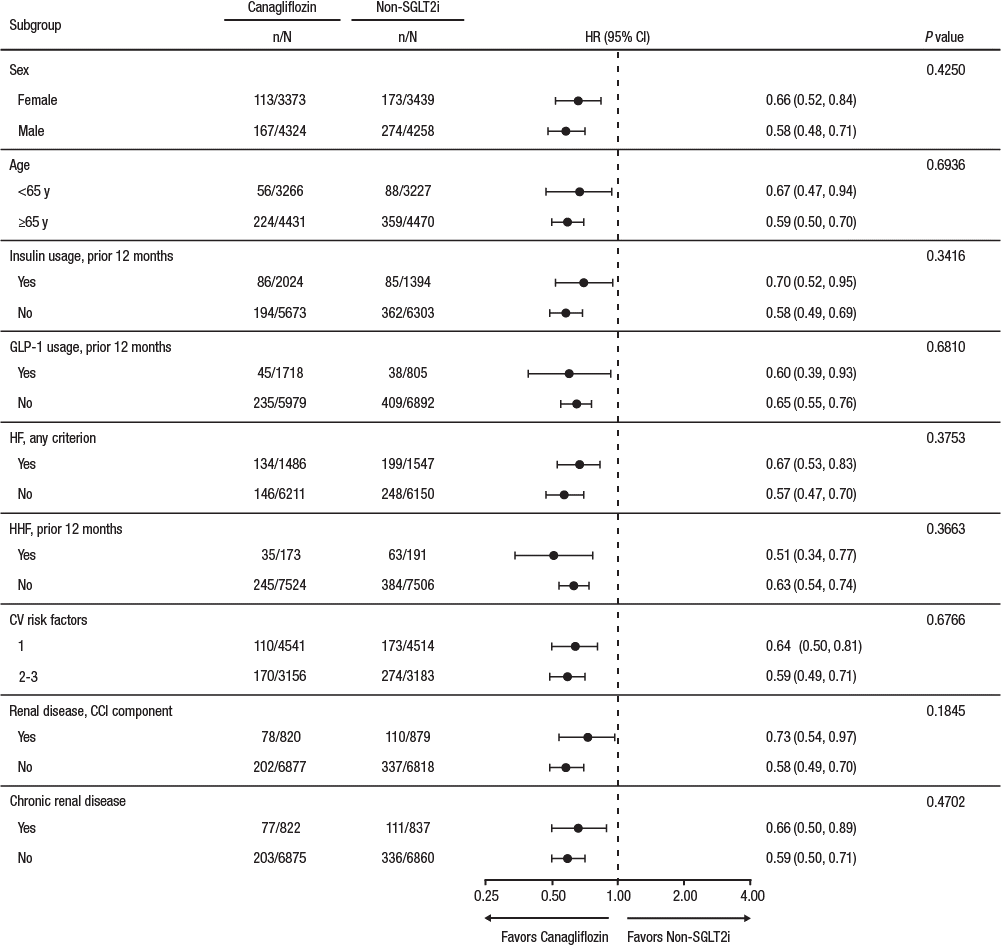


CCI, Charlson Comorbidity Index; CI, confidence interval; CV, cardiovascular; GLP-1, glucagon-like peptide-1; HF, heart failure; HHF, hospitalization for heart failure; HR, hazard ratio; ITT, intent-to-treat; SGLT2i, sodium glucose co-transporter 2 inhibitor.

**Online Figure 5. Sensitivity analysis of the propensity-matched ITT cohort.^†^**

**
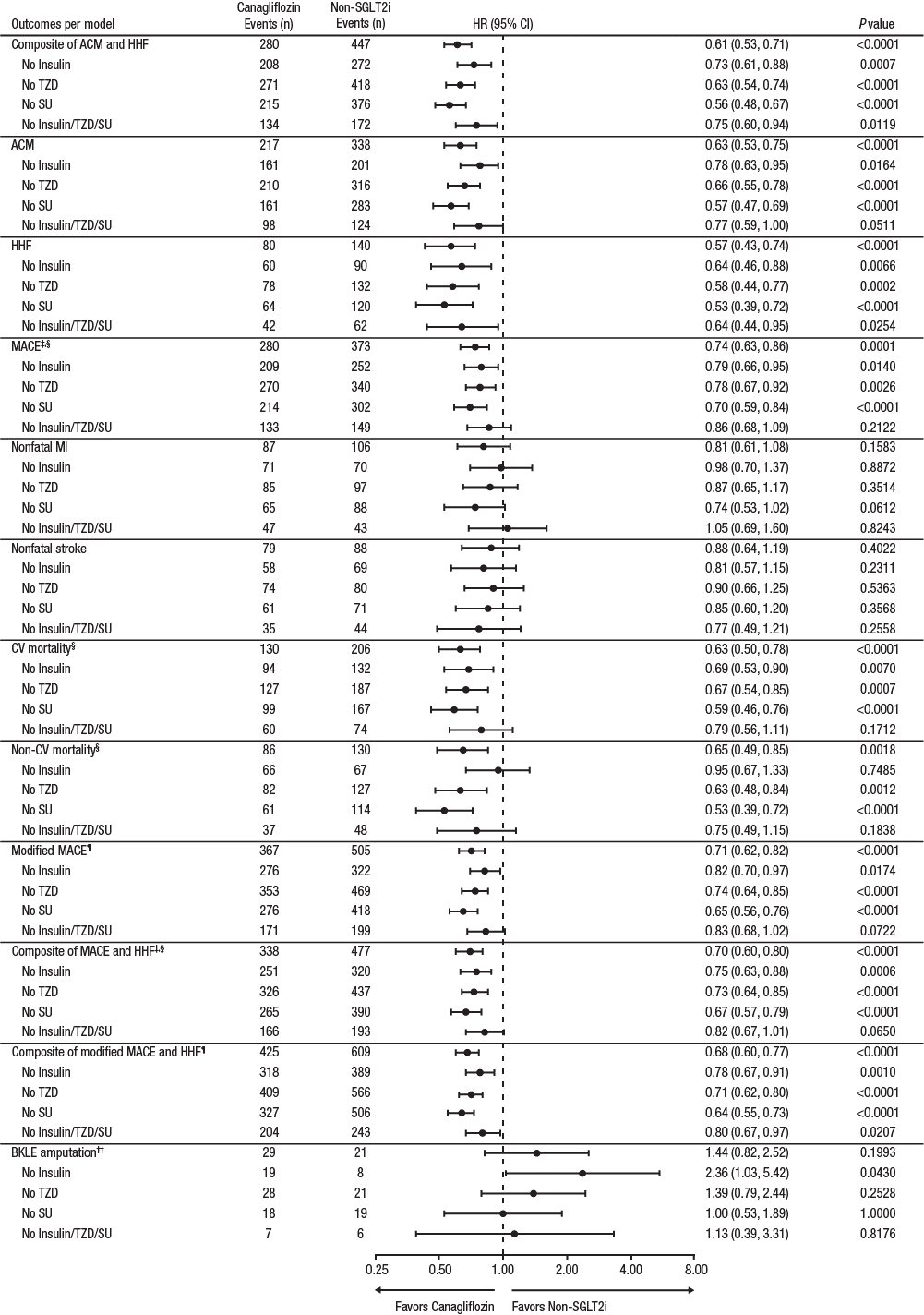
**

ACM, all-cause mortality; BKLE, below-knee lower extremity; CI, confidence interval; CV, cardiovascular; HHF, hospitalization for heart failure; HR, hazard ratio; ITT, intent-to-treat; MACE, major adverse cardiovascular event; MI, myocardial infarction; SGLT2i, sodium glucose co-transporter 2 inhibitor; SU, sulfonylurea; TZD, thiazolidinedione.

^†^Cohort size: primary = 15,394; no insulin = 11,416; no TZD = 14,492; no SU = 12,380; no insulin/TZD/SU = 7,500.

^‡^Patients with an ACM outcome without National Death Index data (n = 3) were excluded from the analysis along with their respective match.

^§^MACE is the composite of CV death, nonfatal MI, and nonfatal stroke.

^¶^Modified MACE is the composite of ACM, nonfatal MI, and nonfatal stroke.

^††^Patients with prior BKLE amputation (n = 6) were excluded from analysis along with their respective match.

**Online Figure 6. Risk of BKLE amputation in the propensity-matched ITT cohort stratified by treatment status and baseline subgroups.^†^**

**
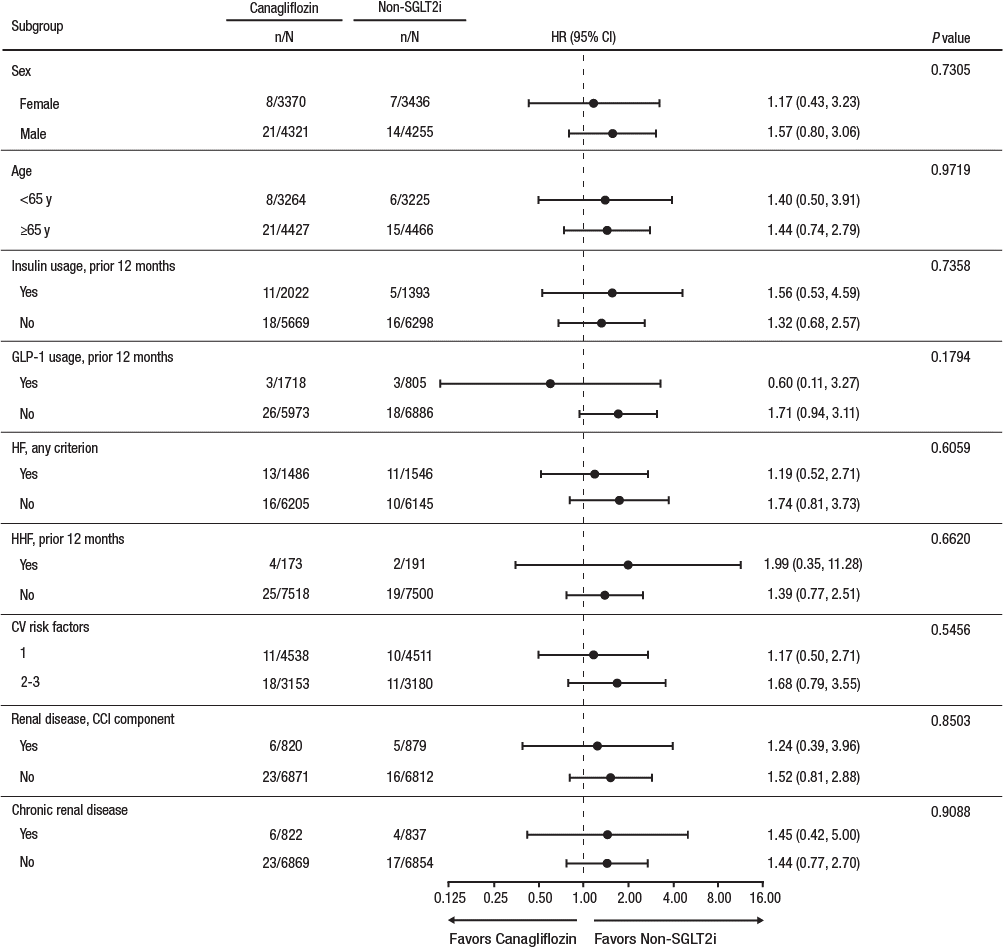
**

BKLE, below-knee lower-extremity; CCI, Charlson Comorbidity Index; CI, confidence interval; CV, cardiovascular; GLP-1, glucagon-like peptide-1; HF, heart failure; HHF, hospitalization for heart failure; HR, hazard ratio; ITT, intent-to-treat; SGLT2i, sodium glucose co-transporter 2 inhibitor.

**^†^**Patients with prior BKLE amputation (n = 6) were excluded from analysis along with their respective match.
